# Telehealth Treatment for Opioid Use Disorder During Pregnancy

**DOI:** 10.1001/jamanetworkopen.2024.2463

**Published:** 2024-03-14

**Authors:** M. Justin Coffey, Maxwell Weng, Cynthia Jimes, Shannon Brigham, Marlene C. Lira

**Affiliations:** 1 Workit Labs, Workit Health, Ann Arbor, Michigan; 2 Geisinger Commonwealth School of Medicine, Scranton, Pennsylvania; 3 David Geffen School of Medicine at University of California Los Angeles, Los Angeles, California; 4 DrPH Program, Bloomberg School of Public Health, Johns Hopkins University, Baltimore, Maryland

## Abstract

This cohort study evaluates opioid use disorder (OUD) treatment and pregnancy outcomes among pregnant patients receiving OUD care through a multistate telemedicine program in the US.

## Introduction

Opioid use disorder (OUD) contributes to high rates of maternal morbidity and mortality in the United States.^1^ Although treatment with buprenorphine during pregnancy is safe, effective, and reduces maternal mortality, pregnant people face barriers to treatment.^2,3^ Telehealth has emerged as a promising modality for delivering and increasing access to OUD care,^4,5,6^ yet outcomes for pregnant populations within dedicated telehealth settings have not been reported.

## Methods

This cohort study evaluated OUD treatment and pregnancy outcomes among pregnant patients receiving OUD care through a low-barrier, multistate telemedicine addiction treatment program in the US. The study was approved by an external institutional review board (IRB), Solutions IRB, and included a waiver of informed consent. This report follows the Strengthening the Reporting of Observational Studies in Epidemiology (STROBE) reporting guideline for cohort studies.

The clinical care model has been described previously.^4^ Electronic medical record data were used to identify patients who were aged 18 years or older, were diagnosed with OUD, received buprenorphine or buprenorphine and naloxone treatment, and had documentation of self-reported pregnancy in problem lists between January 1, 2018, and December 31, 2022. Data were collected through medical record review and analyzed between December 2022 and September 2023. The primary outcome was continuous OUD care through pregnancy vs discharge due to loss to follow-up or administrative or financial reasons. Secondary outcomes included continued telehealth treatment vs transfer of care to a prenatal clinician, retention in care through 6 weeks’ postpregnancy, treatment adherence through urine drug screen confirmation of buprenorphine, and obstetric outcome. Descriptive statistics characterized patients overall. Fisher exact tests, *t* tests, and χ^2^ tests were conducted using Stata version 15 (StataCorp) to assess unadjusted differences between individuals with and without continuous OUD care.

## Results

Ninety-four individuals met inclusion criteria. The mean (SD) age was 32.3 (5.4) years, 74 patients (78.7%) received Medicaid, and 21 patients (22.3%) lived in rural zip codes. Other substance use and mental health conditions were prevalent (Table). Mean (SD) gestational age at disclosure of pregnancy was 14.6 (9.2) weeks. Forty patients (42.6%) were pregnant during their initial telehealth OUD appointment, while 54 (57.4%) became pregnant after establishing care.

**Table.** Characteristics of Pregnant Individuals Receiving Telehealth Treatment for Opioid Use Disorder, Overall and by Continuous Care Through Pregnancy, 2018-2023

Characteristics	Participant, No. (%)			*P* value
	Overall	Continuous OUD care through pregnancy	Lost to follow-up or discharged due to administrative or financial reasons	
No. (%)	94 (100.0)	75 (79.8)	19 (20.2)	NA
Age, mean (SD), y	32.3 (5.4)	32.6 (5.5)	31.3 (5.1)	.36
Sex				
Female	94 (100.0)	75 (100.0)	19 (100.0)	NA
Male	0	0	0	
Pregnant at first telehealth appointment	40 (42.6)	28 (37.3)	12 (63.2)	.04
Gestational age at disclosure, mean (SD), wk	14.6 (9.2)	14.1 (8.6)	16.7 (11.5)	.29
Buprenorphine formulation				
Buprenorphine and naloxone	84 (89.4)	66 (88.0)	18 (94.7)	.39
Buprenorphine	10 (10.6)	9 (12.0)	1 (5.3)	
Mental health diagnoses				
Depression	49 (52.1)	42 (56.0)	7 (36.8)	.14
Anxiety	57 (60.6)	47 (62.7)	10 (52.6)	.42
PTSD	14 (14.9)	10 (13.3)	4 (21.1)	.47
Concurrent substance use				
Nicotine use	63 (67.0)	48 (64.0)	15 (78.9)	.22
Cannabis use	27 (28.7)	22 (29.3)	5 (26.3)	>.99
Illicit stimulant use	18 (19.1)	13 (17.3)	5 (26.3)	.37
Illicit benzodiazepine use	8 (8.5)	6 (8.0)	2 (10.5)	.66
Insurance type				
Commercial	10 (10.6)	7 (9.3)	3 (15.8)	.62
State Opioid Response Grant	1 (1.1)	1 (1.3)	0	
Medicaid	74 (78.7)	58 (77.3)	16 (84.2)	
Multicoverage	4 (4.3)	4 (5.3)	0	
Uninsured or self-pay	5 (5.3)	5 (6.7)	0	
US Census Region				
Northeast	15 (16.0)	14 (18.7)	1 (5.3)	.002
Midwest	34 (36.2)	31 (41.3)	3 (15.8)	
South	25 (26.6)	20 (26.7)	5 (26.3)	
West	20 (21.3)	10 (13.3)	10 (52.6)	
Resides in rural area^a^	21 (22.3)	15 (20.0)	6 (31.6)	.28

Abbreviation: PTSD, posttraumatic stress disorder.

^a^ Defined as residing in a zip code with a US Department of Agriculture Rural Urban Commuting Area code of 4 or more.

Seventy-five patients (79.8%) received continuous OUD care throughout pregnancy and 19 (20.2%) did not, including 13 (13.8%) who were lost to follow-up and 6 (6.4%) who were discharged due to administrative or financial reasons. Compared with those who were pregnant at treatment initiation, patients who became pregnant once established in care were more likely to have continuous care (Table). There were also differences in continuous care by region. There were no differences in continuous care by buprenorphine formulation, comorbid substance use, comorbid mental health conditions, or insurance status.

Of those with continuous OUD care, 6 (8.0%) transferred care to prenatal clinicians (Figure). Among the 69 (92.0%) who continued with telehealth, 65 (94.2%) continued care through 6-weeks postpregnancy, and all patients (100%) testing positive for buprenorphine in postpregnancy urine drug screens. Obstetric outcomes for this subgroup were as follows: 52 (82.6%) carried to term, 9 (13.0%) spontaneous termination, and 3 (4.3%) medical termination.

**Figure.**
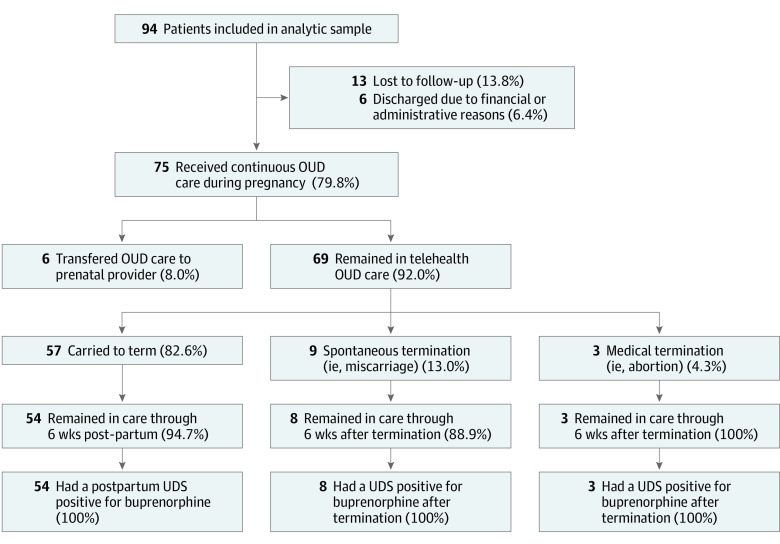
Flow Diagram Depicting Opiod Use Disorder (OUD) and Pregnancy Outcomes Among Pregnant Individuals Receiving Telehealth Treatment for Opioid Use Disorder UDS indicates urine drug screens.

## Discussion

To our knowledge, this study is the first to describe treatment success and obstetric outcomes among pregnant people receiving care through a dedicated telehealth OUD care program. The only comparable study evaluated an integrated care model in which OUD care was delivered within an obstetrics practice. The dedicated telehealth model described here achieved similar treatment outcomes. This study was limited by small sample size; findings may not be generalizable to other settings. Notwithstanding, our findings add to the emerging evidence supporting the effectiveness of telehealth-based OUD treatment in this vulnerable population.

## Notes

Supplement.
Data Sharing Statement
