# Supplementary material for: Telehealth Treatment for Opioid Use Disorder During Pregnancy
Source: JAMA Netw Open. 2024 Mar 14;7(3):e242463. doi: 10.1001/jamanetworkopen.2024.2463 (PMC10940952; doi:10.1001/jamanetworkopen.2024.2463)
Supplement: Supplement. — Data Sharing Statement [file jamanetwopen-e242463-s001.pdf]

Data Sharing Statement

Coffey. Telehealth Treatment for Opioid Use Disorder During Pregnancy. *JAMA Netw Open*. Published March 14, 2024. doi:10.1001/jamanetworkopen.2024.2463

Data
Data available: No
